# A step toward the future? evaluating GenAI QPR simulation training for mental health gatekeepers

**DOI:** 10.3389/fmed.2025.1599900

**Published:** 2025-06-11

**Authors:** Inbar Levkovich, Yuval Haber, Yossi Levi-Belz, Zohar Elyoseph

**Affiliations:** ^1^Faculty of Education, Tel Hai College, Upper Galilee, Israel; ^2^Interdisciplinary Studies Unit, Bar- Ilan University, Ramat Gan, Israel; ^3^Ruppin Academic Center, Lior Tsarfati Mental Pain Center, Emek Hefer, Israel; ^4^Faculty of Eduction, University of Haifa, Mount Carmel, Haifa, Israel

**Keywords:** suicide prevention, gatekeeper training, artificial intelligence, medical simulation, digital health, mental health professionals, primary care, emergency medicine

## Abstract

**Background:**

Suicide remains a leading cause of preventable death, placing a significant burden on healthcare systems worldwide. Effective suicide prevention relies not only on mental health professionals but also on well-trained gatekeepers, including primary care providers, emergency physicians, and community healthcare workers. Traditional training programs, such as *Question, Persuade, and Refer* (QPR), require structured practice and continuous reinforcement to ensure competency. The integration of artificial intelligence (AI)-based simulators into medical training offers a promising, scalable approach for improving suicide prevention skills in healthcare settings. This study evaluates the effectiveness of an AI-driven simulator in enhancing QPR-related competencies.

**Methods:**

A total of 89 adult participants from the community, all of whom were mental health professionals (including social workers, occupational therapists, speech therapists, and physicians), completed pre- and post-intervention assessments measuring self-efficacy and willingness to support individuals at risk of suicide. Participants engaged in real-time interactions with an AI-powered simulator that mimicked conversations with at-risk individuals, enabling dynamic practice of QPR (Question, Persuade, Refer) skills. Data were collected in June 2024. Quantitative data were analyzed using paired *t*-tests and Pearson correlations, while qualitative feedback was examined through content analysis.

**Results:**

Post-intervention self-efficacy scores showed a significant increase, with a large effect size (Cohen’s D = 1.67). Willingness-to-support scores demonstrated a slight but non-significant improvement. Higher QPR self-efficacy correlated positively with increased willingness to support. Qualitative feedback indicated that participants found the simulator realistic and beneficial for skill acquisition, although some expressed concerns regarding the potential reduction of human interaction in mental health training.

**Conclusion:**

AI-driven simulators hold promise as scalable, accessible, and clinically relevant tools for suicide prevention training. Their integration into medical education and clinical settings could improve the preparedness of healthcare providers, primary care physicians, and frontline medical staff in identifying and managing suicide risk. These findings support the adoption of digital health innovations to enhance medical training and public health interventions.

## Introduction

Suicide prevention remains a critical public health challenge, with approximately 817,000 deaths by suicide annually and an estimated 20 million attempts (1). Effective prevention requires diverse strategies, including training gatekeepers, individuals in roles likely to encounter those at risk to assess and respond to suicidal crises (2, 3). Despite its importance, scalable methods for gatekeeper training remain limited. This study examined the efficacy of an artificial intelligence (AI)-based simulator in enhancing gatekeeper competence in suicide risk assessment.

Gatekeeper training equips individuals, such as educators, social workers, and law enforcement with skills to recognize suicide warning signs, initiate supportive dialogue, and refer individuals to mental health services (4). The Question, Persuade, Refer (Question, Persuade, Refer (QPR) method (5) is a widely used, evidence-based training model emphasizing direct questioning, persuasive engagement, and referral to appropriate resources (6). Studies demonstrate QPR’s effectiveness in improving gatekeepers’ knowledge, confidence, and referral rates (2, 7).

However, training effectiveness varies depending on quality, duration, and ongoing support (8). A key limitation is the lack of simulated interventions, hindering practical application. Traditional gatekeeper training is also resource-intensive, requiring periodic refreshers for sustained efficacy (9). AI-based simulators offer a promising solution, enhancing skill acquisition through scalable and interactive role-play scenarios.

Generative artificial intelligence (GenAI), particularly large language models (LLMs), has transformed fields such as education, medicine, and psychology (10, 11). Large language models (LLMs) generate human-like texts based on extensive datasets, enabling applications such as emotion detection, risk assessment, and mental health support (12, 13). While AI’s potential in mental health is significant, ethical concerns persist, including data privacy, algorithmic bias, and overreliance on technology (14, 15). Nevertheless, research indicates that AI systems can assess suicide risk with accuracy comparable to that of mental health professionals and adapt assessments to different cultural contexts (16). Integrating AI into gatekeeper training offers new opportunities for accessible, scalable, and effective interventions. Within healthcare systems, medical professionals are strategically positioned to identify and intervene in cases of suicide risk. Tailoring QPR training to the specific needs of healthcare providers may significantly enhance early detection and intervention efforts in clinical settings (6).

### The current study

Recent research has demonstrated the considerable potential of GenAI for suicide prevention and mental health support. Studies indicate that GenAI systems such as ChatGPT-4 can assess suicide risk with accuracy comparable to that of mental health professionals (10, 11) and can adapt assessments to diverse cultural contexts (12). Beyond risk assessment, GenAI can also facilitate role-playing scenarios for educational and therapeutic purposes, offering a versatile and interactive platform for training and intervention (13–15).

One of the challenges of traditional gatekeeper training is the need for repeated training sessions, extensive resources, intensive practice, and continuous human feedback (8). Integrating GenAI capabilities into role-playing scenarios offers new opportunities for enhancing gatekeeper training for suicide prevention. This integration may also provide immediate and wide-scale access to knowledge about suicide intervention that is adaptable to various users and situations, while creating interactive learning experiences. Specifically, in this study we developed and evaluated an AI-powered QPR-based simulator designed for gatekeeper training in suicide prevention, offering a novel approach to address the limitations of traditional training methods. The simulator utilizes role-play scenarios to provide practical experience in identifying at-risk individuals, asking about suicidal thoughts and intentions, persuading individuals to seek help, and referring them to appropriate resources. While the QPR Institute offers role-play simulations tailored to specific gatekeeper groups, the current simulator was developed independently and does not rely on proprietary QPR Institute models. Its design was guided by publicly available QPR principles and expert input, ensuring both adherence to established guidelines and innovative application through GenAI.

We posited two primary research questions:

1. To what extent can AI-based QPR simulators improve gatekeepers’ self-efficacy and willingness to handle suicide-related situations?
2. How can feedback within an AI-powered QPR simulation tool be optimized to enhance learning outcomes and user experience?

## Methods

### Participants

This study included 89 adult participants recruited from a social media platform dedicated to exploring the intersection between GenAI and responsible mental health care (the “Artificial Third” community). The inclusion criterion was at least 18 years old. Professional mental health practitioners were excluded from the study. In this study, the term “mental health professionals” referred to individuals who had completed introductory training or held paraprofessional roles related to mental health (e.g., social work students, psychology interns, rehabilitation counselors) but were not licensed or fully certified practitioners at the time of participation. Participants were invited to attend a special webinar on June 6, 2024 (via the Zoom platform), during which the simulator was demonstrated, and participants interacted with it online. This interaction included completing a questionnaire that tapped into the main issues of the study. The questionnaire was administered twice: once before interacting with the simulator (pre-measure) and once afterward (post-measure). As part of the demographic section in the pre-intervention questionnaire, participants were asked whether they had previously completed QPR gatekeeper training. The majority (approximately 78%) reported no prior experience with QPR.

### Study design

**FIGURE 1:**
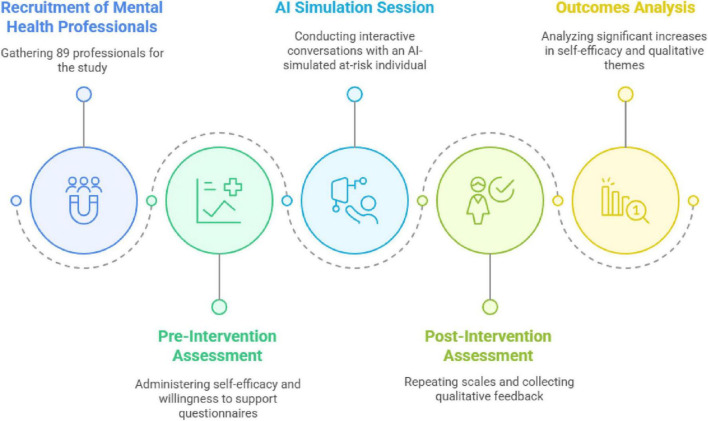
Study flowchart from pre- to post-intervention.

This study integrated quantitative and qualitative approaches to evaluate the effectiveness and ethical considerations in using an AI-powered chatbot for QPR training. As shown in Figure 1, the experimental design included both pre-intervention and post-intervention measurements.

### Intervention

The QPR AI simulator is an innovative tool designed to enhance gatekeeper training to help someone experiencing a suicidal crisis. The AI-Bot was built by the authors between March and June 2024. Its operation relied exclusively on a detailed prompt architecture engineered by the research team to guide the base AI model (GPT-4o), without any model fine-tuning or external data integration (e.g., Retrieval-Augmented Generation, RAG). Interaction with the bot entailed three key stages. First, each user was introduced to the simulation’s objective and structure to provide a clear framework for the interactive learning experience. All participants were explicitly told that both the simulation and the feedback were generated by GenAI, and they gave their informed consent to participate. Second, each user was invited to engage in conversation with one of two unique AI-generated characters via the chat interface. Each character represented a distinct scenario (man or woman with a unique case story, communication style, and risk factor profile) with behaviors and responses governed by the prompt architecture.

Finally, the bot offered detailed constructive feedback generated through an automated analysis of the interaction, governed by the same core prompt architecture. This analysis evaluated the participant’s performance against several key criteria defined within the prompt. These criteria included the participant’s effectiveness in building rapport (demonstrating empathy and trust), their ability to identify relevant risk and protective factors, and critically, their application of the QPR model’s core components. QPR has three core components: (1) Question: directly inquiring about suicidal thoughts or intentions to emphasize the importance of breaking the silence surrounding suicide. (2) Persuade: encouraging at-risk individuals to seek professional help while leveraging the interpersonal connections established through questioning. (3) Refer: facilitating access to appropriate mental health resources or crisis intervention services. The AI-Bot assessed how skilfully each of these QPR components was implemented during the dialogue. Following this multi-faceted evaluation, the AI-Bot generated structured qualitative feedback addressing the assessed areas (rapport, risk assessment, QPR application) with specific examples from the conversation, and provided an overall assessment with suggestions for improvement. Additionally, a quantitative score (1–9, 16) was assigned, calculated using an embedded scoring rubric within the prompt that reflected the participant’s overall demonstrated competency across all evaluated criteria.

To enhance transparency regarding the prompt’s construction and support potential research replication, Table 1 summarizes the key components of this prompt architecture. Researchers interested in obtaining the full prompt for the specific purpose of academic replication may contact the corresponding author. Access will be considered subject to agreement on terms of use, as detailed in the note below Table 1.

**TABLE 1:** Architecture of the GenAI QPR simulation prompt.

Stage/component	Purpose	Key elements and directives implemented in prompt
1. Initialization and setup	Define AI role, establish simulation context, inform user about the process, rules, and feedback mechanism.	Defined AI/user roles, simulation objectives, interaction rules (e.g., non-verbal cues, response limits), and the automated feedback procedure. Included mandatory introductory message and AI disclaimer.
2. Character simulation	Embody the at-risk individual realistically based on a predefined profile, interact dynamically with the user.	Instructed AI to embody persona based on the embedded character profile (derived from Appendix A within the prompt). Included directives for realistic interaction based on this profile (e.g., gradual disclosure, resistance points).
3. Simulation management and flow control	Control the initiation, progression, and termination of the simulation interaction.	Implemented logic for simulation initiation, turn tracking, termination detection (user command or turn limit), and controlled transition between AI personas.
4. Automated feedback generation	Provide structured, critical, and actionable feedback on the user’s QPR performance based on the interaction.	Defined the feedback expert AI persona and feedback structure. Instructed AI to evaluate user performance against QPR principles (guided by embedded QPR Principles in the prompt’s), assess rapport-building, identify risk/protective factors, and provide overall assessment. Mandated grounding feedback in conversational examples and applying an embedded scoring rubric (1–9, 16). The rubric linked specific score ranges to performance levels: severe failures/unethical conduct (1, 2), significant gaps/inconsistent skills (3–6), solid competency (7, 8), and excellent/exemplary application (9, 16) Included AI limitations disclaimer.
5. Knowledge base integration	Provide the foundational information necessary for the AI to perform its simulation and feedback roles accurately.	Embedded two key knowledge artifacts within the prompt: Character Profile: Included character’s background context, specific communication style directives and core psychological concerns/themes (e.g., loneliness, worthlessness, hopelessness) QPR Principles and Guidelines Summary: Included definitions, risk/protective factors, warning signs recognition, and specific ‘Dos and Don’ts’ for communication during each QPR stage (Question, Persuade, Refer), forming the basis for simulation logic and feedback evaluation.

The full prompt, including internal logic and evaluation criteria, is available upon request from the corresponding author due to ethical considerations.

Table 1 summarizes the key components of this prompt architecture. Qualified researchers may request access to the full prompt under specific conditions (Table 1).

The simulator was tested and validated by three mental health experts specializing in suicide prevention. To mitigate potential biases related to age, gender, and race, the simulator’s character scenarios and prompts were carefully designed using neutral and inclusive language. The research team conducted preliminary evaluations of the model’s outputs, systematically reviewing and adjusting the prompts and AI responses to avoid the reinforcement of stereotypes. Participants were explicitly informed that the simulator was not intended to fully capture cultural sensitivities, and they were encouraged to report any perceived biases during their interaction. These proactive measures aimed to enhance the model’s fairness and inclusivity.

### Measures

#### Pre- and post-intervention questionnaire

The following two subscales, QPR Self-Efficacy and Willingness to Support, were administered both before and after the intervention to assess changes in participants’ perceived ability and motivation to support individuals undergoing a suicidal crisis. Using the same items in both the pre- and post-intervention phases enabled a direct comparison of participants’ self-assessments over time.

*QPR Self-Efficacy* (14)- This subscale measured participants’ perceived competence in responding to a suicidal crisis, based on the principles of the QPR (Question, Persuade, Refer) model. Each item was rated on a 10-point Likert-type scale ranging from 1 (“not at all”) to 10 (“completely”).

It included the following two items: To what extent do you feel capable of supporting people undergoing a suicidal crisis; To what extent do you feel you have the tools to support a person undergoing a suicidal crisis. The responses were averaged to create a composite self-efficacy score. The scale demonstrated excellent internal consistency (Cronbach’s alpha = 0.97).

*Willingness to Support* (14)- This subscale assessed participants’ motivation and readiness to engage in helping behaviors toward individuals at risk of suicide. Each item was rated on a 10-point Likert-type scale ranging from 1 (“not at all”) to 10 (“completely”). It included the following two items: If given the opportunity, how willing would you be to support a person undergoing a suicidal crisis; If you were asked to assist a person in dealing with a suicidal crisis, to what extent would you want to try and help. The responses were averaged to create a composite score for willingness to support. This subscale showed strong internal reliability (Cronbach’s alpha = 0.89).

#### Post-intervention questionnaire

Following the simulation experience, participants completed a post-intervention questionnaire designed to evaluate their experiences and attitudes. This questionnaire included four items identical to those used in the pre-intervention phase—two assessing QPR self-efficacy (Cronbach’s alpha = 0.86) and two assessing willingness to support a person undergoing a suicidal crisis (Cronbach’s alpha = 0.90). This allowed for a direct comparison of participants’ responses before and after the intervention.

In addition, four new items were included to assess participants’ perceptions of the simulator experience:

To what extent did your experience with the simulator prepare you to help someone coping with a suicidal crisis; How much do you feel you learned from your experiences; To what extent do you feel your feedback will help you provide assistance to a person undergoing a suicidal crisis; Would you recommend this AI-based simulator experience to others before assisting someone at risk.

The questionnaire also included four open-ended qualitative questions designed to gather deeper insights into participants’ subjective experiences. These questions aimed to explore participants’ reflections on the learning process, perceived strengths and limitations of the simulator, and considerations for future development. The questions were as follows:

How would you evaluate your learning experience with an AI-based simulator; What do you suggest for improving the AI simulator; What advantages do you find in this training program; What risks or concerns do you have about this type of training.

These qualitative responses provided valuable context and depth to the quantitative findings, allowing for a more comprehensive understanding of the simulator’s impact.

### Procedure

The study was conducted during a live webinar (online conference) initiated by the authors. Participation was voluntary. All questionnaires, including both the pre-intervention and post-intervention measures as well as the demographic questions and open qualitative items, were administered through Google Forms. An explanation of the bot was provided during the webinar. Interested participants were sent links to the questionnaires and the AI-based simulator via Zoom chat. Subsequently, they were asked to interact with one of the two selected bots (male or female). Following this interaction, participants responded to a post-intervention questionnaire. Interaction with the AI-based simulator took approximately 15-25 min. Participants communicated with the bots from their homes. They were instructed to interact using a computer rather than a mobile device.

This study was approved by the university Ethics Committee (Institutional Review Board Approval Number: 2024-67 YVC EMEK). Participants were fully informed of the study’s aims and procedures and were told they could withdraw at any stage without any repercussions. They were assured of the confidentiality and anonymity of their data and were instructed not to enter any personal information during their interaction with the simulator.

All conversations were recorded for research purposes, with the informed consent of the participants. Supplementary Appendix A describes one complete conversation. The data were securely stored and managed by the current research team to ensure participants’ privacy and confidentiality. The simulator was powered by GPT-4o (OpenAI Ltd.), selected as it was considered the most advanced publicly available LLM via API at the time, offering sophisticated and multimodal capabilities and improved speed for enhanced user experience. Access to this simulation system is facilitated through API technology, allowing seamless integration with external platforms. The AI bot was deployed using PMFM AI, an innovative application that specializes in making AI-powered chatbots accessible to the public. As detailed in the Intervention section, the AI operated solely based on the prompt architecture without model fine-tuning or RAG techniques. The entire simulation and data processing occurred within the secure cloud-based environments of these platforms.

### Data analysis

Descriptive statistics were used to describe the participants’ demographic baseline data. After that, a series of paired *t*-tests compared the participants’ responses on the study measures before and after the intervention. Pearson’s correlations were calculated to examine the relationships between the background data and the dependent variables.

In addition to parametric analyses (*t*-tests, Pearson correlations), we conducted non-parametric tests (Wilcoxon signed-rank, Spearman correlations) to address potential concerns regarding the ordinal nature of Likert-scale data. The criterion for determining statistical significance throughout the study was set at *p* < 0.05. All quantitative analyses were conducted using SPSS (v. 27). Qualitative data were analyzed using content analysis.

### Qualitative analysis

The qualitative data were analyzed using conventional content analysis, following three stages: open, axial, and selective coding. In the first stage (open coding), two independent coders carefully read all participant responses multiple times to achieve immersion and independently identified initial codes directly from the data. In the second stage (axial coding), the coders grouped similar codes into broader conceptual categories by identifying relationships and connections between the codes. In the third stage (selective coding), overarching themes were developed, integrating the categories into coherent thematic structures that captured the main aspects of participants’ experiences. Discrepancies between coders were resolved through discussion until full consensus was achieved. This analytic process ensured both inductive theme generation and systematic validation of the emerging categories and themes.

## Results

### Study sample

The study included 89 participants ranging in age from 26 to 72 years (Mage = 37.69, SD = 13.82). The sample’s gender distribution was predominantly female (*n* = 74, 83.15%). A substantial portion of participants were mental health professionals, including social workers, occupational therapists, speech therapists, and physicians.

### Impact of intervention on participants’ self-efficacy

**FIGURE 2:**
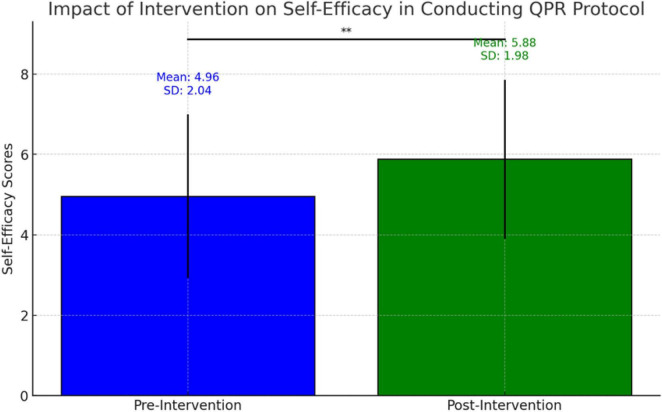
Self-efficacy in assisting at-risk individual, pre- and post-AI simulator intervention (*N* = 89). This graph illustrates the participants’ self-efficacy in assisting a person in suicidal distress before and after using the AI simulator. The blue bar represents the mean pre-intervention self-efficacy score (pre) and the green bar represents the mean post-intervention self-efficacy score (post). *0.05.

A paired sample *t*-test was conducted to evaluate the impact of the intervention on participants’ self-efficacy in conducting the QPR protocol. As shown in Figure 2, self-efficacy scores on an 11-point scale increased significantly from pre-intervention (*M* = 4.96, *SD* = 2.04) to post-intervention (*M* = 5.88, *SD* = 1.98), *t*(88) = −5.17, *p* = 0.001 (two-tailed). This finding indicates that participants believed they were more capable of conducting the QPR protocol following the intervention. The effect size was high (Cohen’s D = 1.673683). Additional non-parametric analysis using the Wilcoxon signed-rank test also confirmed a statistically significant difference (*Z* = −4.983, *p* < 0.001), strengthening the validity of the findings regardless of the statistical analysis approach.

### Impact of intervention on willingness to support

A paired-sample *t-*test was conducted to evaluate the impact of the intervention on participants’ willingness to help at-risk individuals. The willingness-to-support scores increased slightly from pre-intervention (6.79 ± 2.15) to post-intervention (6.89 ± 2.15), but the difference was not significant (t (88) = −0.67, *p* = 0.50).

### Correlation analysis

A positive correlation emerged between improvement in QPR self-efficacy and increased willingness to support. As participants began to feel more capable, they became more willing to extend help. No significant relationships were found between age or professional experience and improvements in perceived self-efficacy or willingness to support.

### Simulator use experience

**FIGURE 3:**
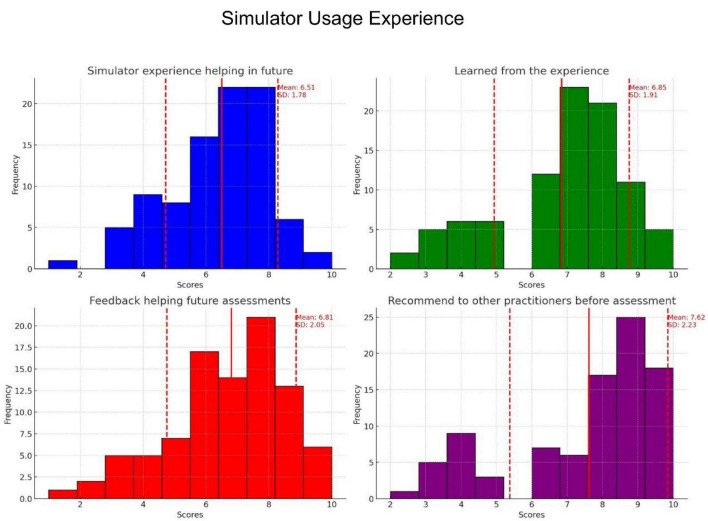
Distribution of participants’ responses to four questions regarding their experiences with the AI simulator: To what extent will the simulator experience help you support a person in suicidal distress in the future (blue)? How much do you feel you learned from your experience (green)? How much feedback do you feel would help you in future assistance situations (red)? Would you recommend this AI-based simulation experience to other practitioners before helping a person in suicidal distress (purple)? Each histogram illustrates the frequency of the participants’ scores, with the red lines indicating the means and standard deviations.

As illustrated in Figure 3, most participants reported positive learning outcomes after using the simulator. On the question regarding the extent to which the simulator helped them conduct future assessments, the mean score was 6.51 ± 1.78. On the question of how much participants felt they had learned from the experience; the mean score was 6.85 ± 1.91. Regarding how helpful the feedback was for future assessments; the mean score was 6.81 ± 2.05. Lastly, for recommendation to other practitioners, the mean score was 7.62 ± 2.23. These results suggest that participants generally found the simulator beneficial and were likely to recommend it to others.

#### Qualitative analysis of simulator feedback

Three themes emerged from participants’ responses: realism and engagement, concerns about human connection, and empowerment through practice.

### Theme 1: realism and emotional engagement

Many participants were struck by the simulator’s authenticity, describing it as “fascinating” and “intellectually challenging.” Some noted the emotional depth, with one stating, “The interaction was very real, almost disturbing in its accuracy.” The immersive nature fostered psychological safety, allowing users to practice without fear of real-world consequences. As one participant put it, “It’s educational and liberating, you can make mistakes without harming anyone.” Others valued its accessibility, reporting that it reduced shame and pressure compared to traditional training.

### Theme 2: balancing AI benefits and limitations

While participants acknowledged the benefits of AI-based training, some expressed concern about its limitations in replicating human interaction. One participant noted, “There is no way to express my personal approach; the responses feel pre-scripted.” Others suggested a hybrid model combining AI simulations with instructor-led discussions. Additional concerns included data privacy, reliability of AI-generated feedback, and legal implications, with one participant asking, “If I fail to prevent suicide, can I be held liable?” Others questioned whether AI models perpetuate biases in mental health, citing experiences where AI-generated content appeared stigmatizing.

Some participants noted that certain responses felt overly generic or lacked cultural sensitivity. One participant commented, “It felt like the bot didn’t really understand where I was coming from it gave a standard answer that didn’t fit my background.” Such impressions led a few participants to raise broader concerns about whether AI tools adequately reflect the diversity of human experiences.

### Theme 3: empowerment through practice

Despite concerns, many participants reported increased confidence and preparedness. One shared, “It helped me regain my belief that I can intervene and make a difference.” The non-judgmental learning environment was especially valued, as it allowed users to practice suicide prevention skills in a low risk setting. Participants also highlighted its effectiveness in teaching communication strategies, such as how to ask about suicidal thoughts and create a supportive dialogue. One noted, “It’s a way to learn that stays with you, something you’ll remember if you ever need it.”

## Discussion

### Quantitative findings: self-efficacy and willingness to support

This study evaluated the efficacy of an AI-based simulator in enhancing gatekeeper competence and willingness to provide support during suicide prevention training. Participants reported increased self-efficacy in applying the QPR model after engaging with the AI-based simulator, a finding that aligns with previous research on gatekeeper training effectiveness. Similar studies have also documented improved confidence among gatekeepers following QPR training (2, 6, 7). Several factors may explain this increase in self-efficacy. First, the AI simulator provided a safe, low-stake environment for practice, enabling participants to engage in realistic scenarios without fear of real-world consequences. This finding aligns with Bandura’s (17) self-efficacy theory, which posits that mastery experience is crucial for building confidence. Second, the immediate, personalized feedback offered by AI likely contributed to the participants’ sense of competence, which is essential for skill retention in gatekeeper training (18). Additionally, the high fidelity of AI-simulated interactions, as noted in our qualitative findings, may have enhanced the perceived transferability of skills to real-world situations, further boosting self-efficacy.

Although willingness-to-support scores showed a slight increase from pre-intervention to post-intervention, the difference was not statistically significant. This result suggests either the absence of a meaningful effect or that the study may have lacked sufficient statistical power to detect smaller changes, considering the limited sample size and short time frame between intervention and assessment. Previous research similarly noted that improvements in attitudes or behavioral intentions following gatekeeper training can be variable and may require longer-term reinforcement to become significant (8, 9). Therefore, future studies should consider larger samples and extended follow-up periods to better evaluate changes in willingness to intervene.

Several factors may explain this modest and non-significant increase in willingness to treat. The willingness to intervene in suicidal crises is influenced by a complex interplay of factors beyond mere skill acquisition, among them personal beliefs, cultural attitudes, and perceived social support (3). Additionally, the baseline willingness-to-treat scores in our study were relatively high, suggesting a potential ceiling effect on significant improvement. Moreover, the short duration between the intervention and post-test may not have allowed sufficient time for participants to fully process their experiences and recalibrate their willingness to intervene. Changes in behavioral intentions often require more time and real-world reinforcement to manifest significantly (4). Furthermore, the realistic nature of the AI simulation may have heightened participants’ awareness of the challenges involved in suicide prevention, potentially tempering any immediate increase in willingness to treat.

### Qualitative insights and the role of the learning experience

The positive user experience reported by the study participants aligns with emerging research on the acceptability and perceived utility of AI-based training tools in mental health education (13). The high mean scores across various aspects of simulator experience echo findings from similar studies on virtual simulation in healthcare education (19, 20). The qualitative research results suggest deeper explanations for these findings. The realism of simulated interactions- a key theme in our qualitative data-likely contributed to the high ratings of perceived learning and preparedness for future assessments. This aligns with the concept of “presence” in virtual learning environments, identified as a crucial factor in effective training (21). Additionally, the safe, non-judgmental learning environment provided by the simulator likely contributed to the high recommendation rates. This finding emphasizes the importance of psychological safety in effective gatekeeping training (6). The ability to practice sensitive conversations without real-world consequences- a benefit noted by several participants- addresses a key challenge in traditional suicide prevention training methods (8).

Moreover, the immediate, personalized feedback provided by AI likely enhanced its perceived educational value. This aligns with best practices in simulation-based learning and the importance of timely, specific feedback in maximizing learning outcomes (16). The convergence of quantitative ratings and qualitative themes provides a robust, multifaceted picture of the user experience. The quantitative data offered a broad overview of user satisfaction, whereas the qualitative insights provided depth and context, explaining why participants found the simulator beneficial. Note, however, that despite the overall positive reception, some participants expressed concerns about the potential erosion of human connections in mental health training, as revealed by our qualitative analysis. This tension between technological advancement and the need for human touch in mental health education suggests areas for future refinement of AI-based training tools (22).

As a pioneering study in AI-based suicide prevention training, our research prompts significant questions about the future of gatekeeper education. Despite the immediate benefits in self-efficacy, the use of AI as an “artificial third” (23, 24) introduces complex ethical and professional considerations. Consistent AI-generated feedback may influence gatekeepers’ self-efficacy and knowledge development in ways not yet fully understood, raising concerns about the potential loss of human nuances essential to suicide prevention. The introduction of AI into the traditional trainer-trainee dynamic could reshape professional development in mental health interventions (13–15). This novel approach requires careful ethical consideration, especially regarding AI’s ability to provide empathetic and contextually appropriate feedback in this sensitive area. Despite the promising results, our study highlights the critical need to balance technological advancements with the preservation of essential human elements in training.

### Study implementations

In the context of implementation within healthcare systems, the simulator offers several unique advantages. First, the ability to adapt the simulation to specific clinical environments (emergency departments, primary care clinics) enables context-focused training. Second, the capacity to repeatedly practice communication skills through AI aligns with contemporary models of continuous learning in medical education (25). Finally, in an era where digital health has become an integral part of healthcare systems, incorporating AI tools in training better prepares healthcare professionals for the future of medicine (26). Nevertheless, it should be emphasized that the simulator is designed to complement, not replace, human intervention in clinical training and support (27).

### Limitations

This study has several limitations. The small, predominantly female sample limits generalizability, and self-reported measures may introduce bias, not necessarily reflecting real-world behavior. The short interval between intervention and assessment prevents conclusions about long-term skill retention. The study also lacked data on participants’ prior knowledge, personal, and cultural factors, which may have influenced responses. The absence of a control group further restricts causal inferences. While qualitative feedback was insightful, selection bias remains a concern, as noted in similar AI-based intervention studies (28). Additionally, although efforts were made to minimize biases in the AI model, the possibility of subtle algorithmic biases affecting user interactions cannot be entirely ruled out. Furthermore, the lack of comparable studies using identical QPR outcome measures with human-only instructors precludes direct benchmarking of effect sizes—this notable limitation has also been acknowledged. Future research should include larger, more diverse samples, objective measures, follow-ups, and randomized controlled trials to assess AI-based simulator efficacy more rigorously.

## Conclusion

This study assessed an AI-based simulator’s effectiveness in QPR gatekeeper training. Findings showed a significant increase in self-efficacy, while willingness to support saw a slight, non-significant rise. Qualitative feedback highlighted realism and engagement but raised concerns about reduced human interaction. AI simulators can complement traditional training by providing a safe, accessible learning tool. Recommendations include integrating AI for skill enhancement, regular practice, self-care, and peer discussions to address AI limitations. Gatekeepers should remain flexible, empathetic, and culturally aware, as simulations may not fully capture real-world complexities. From a clinical perspective, integrating AI-based simulators in training healthcare professionals to identify and intervene in cases of suicide risk could promote earlier and more effective interventions. Medical schools, hospitals, and community clinics should consider adopting these tools as part of training and residency programs, particularly given the growing need for remote training and flexibility in continuing medical education. Future research should examine long-term impact on clinical outcomes and rates of identification and referral in medical settings.

## Data Availability

The raw data supporting the conclusions of this article will be made available by the authors, without undue reservation.
