# Supplementary material for: A step toward the future? evaluating GenAI QPR simulation training for mental health gatekeepers
Source: Front Med (Lausanne). 2025 Jun 11;12:1599900. doi: 10.3389/fmed.2025.1599900 (PMC12187690; doi:10.3389/fmed.2025.1599900)
Supplement: Supplementary file 1 [file Table_1.docx]

# Appendix A: Documentation of Jacob’s (Pseudonym) Gatekeeper Simulation Using the QPR Model with Ido, an AI-Based Character

| **AI** | **User (Jacob)** | **Stage Number** |
| --- | --- | --- |
| **AI**: Hello! I am an AI-based simulator designed to help you practice using the QPR model (Question, Persuade, Refer) for suicide prevention as a community gatekeeper. In this simulation, you will play the role of a colleague in a high-tech company who is having a conversation with Ido, a 29-year-old reservist who recently returned from duty in Gaza. Although you and Ido are not close friends, you noticed that he seems strange and withdrawn since his return. You decided to talk to him and ask how he is doing.  During the simulation, I will convey Ido's emotional state and non-verbal responses by writing in square brackets []. I would appreciate it if you could also write any non-verbal communication in square brackets [], if necessary.  You can end the simulation by typing 'end'. I will monitor each of my responses. Once I reach 45 responses, the conversation will automatically end.  After you type 'end', I will step out of Ido's role and adopt the role of Rotem, an expert psychologist, who will provide you feedback on your use of QPR skills during the conversation with Ido. She will assess how you:   - Asked directly about suicidal thoughts (Question) - Listened with empathy, offered hope, and encouraged seeking help (Persuade) - Recommended professional support (Refer)   Please note that although I will realistically portray Ido, I am an AI model and not a real person.  Are you ready to begin the simulation? Once you agree, I will start the conversation in the role of Ido.  Before we begin, please provide the last 5 digits of your ID number. This information is collected solely for cross-referencing with research questionnaires and will not be used for any other purpose. We are committed to maintaining participants’ anonymity in the research and the simulation. | **Jacob**: Hi | Stage A- Consent & Simulation setting |
| **AI (as Ido)**: Thank you very much. Let's begin the simulation.  ----- [Ido enters the room, sits on the couch, and looks at the floor.] "Hey..." [avoids eye contact, slouches on the couch] | **Jacob**: XXXXX | Stage B- Simulation |
| **AI (as Ido)**: [Ido glances at you briefly before looking away]. "Hey..." [still avoids eye contact] | **Jacob**: Hi |  |
| **AI (as Ido)**: [Ido shrugs, still avoiding eye contact] "I'm fine... How are you?" | **Jacob**: How are you? |  |
| **AI (as Ido)**: [Ido fidgets slightly, seeming uncomfortable] "Yeah, I've just been busy... work and all..." [forces a small smile, but it doesn’t reach his eyes] | **Jacob**: I'm fine, bro. How are you? We haven't talked in a long time |  |
| **AI (as Ido)**: [Ido's face tenses up, and he shifts uncomfortably] "I'm managing... I don't really want to talk about it right now." [avoids eye contact, looks visibly stressed] | **Jacob**: Since you came back from the reserves, I've felt that you're a bit down. How are you? It must not be easy to return. |  |
| **AI (as Ido)**: [Ido nods slightly, still looking at the floor] "Yeah, it's really not easy... but it is what it is." [sighs deeply, appears resigned] | **Jacob**: I have another friend who came back, I know it's not easy, bro. |  |
| **AI (as Ido)**: [Ido takes a deep breath, still looking down] "It's... a bit difficult. Harel and I are arguing all the time, and the kids... they just don't understand." [shoulders sag, looks exhausted] | **Jacob**: How are things at home? |  |
| **AI (as Ido)**: [Ido hesitates, frowning] "About everything... about how I don't help enough at home, about how I'm not focused. She says I'm not the same person I was before I went to the reserves." [voice shakes slightly, eyes still avoidant] | **Jacob**: What are you arguing about? |  |
| **AI (as Ido)**: [Ido pauses, taking a moment to gather his thoughts] "Yeah... I guess I do. I feel a little lost, like I'm not really here." [voice is weary, eyes downcast] | **Jacob**: Do you feel that way too? |  |
| **AI (as Ido)** [Ido stiffens slightly, eyes narrowing] "Yes, it was terrible. I lost a close friend there... he was killed, and I couldn't do anything." [voice breaks slightly, filled with guilt]. | **Jacob**: Sounds tough, bro. Was it hard there? |  |
| **AI (as Ido)**: [Ido's eyes well up with tears, but he quickly wipes them away] "I don't know about that... sometimes it feels like I really missed the mark. Maybe I could have saved him." [voice choked, guilt evident] | **Jacob**: I'm so sorry. I'm sure you did everything you could. |  |
| **AI (as Ido)**: [Ido nods slightly, though his eyes remain downcast] "Yes, I understand... but it doesn't make it any easier. This feeling of helplessness just won't leave me." [voice filled with despair, shoulders slumped] | **Jacob**: I can understand this is how you feel. But you know, this is a feeling many people experience in this situation—a feeling of worthlessness and sadness |  |
| **AI (as Ido)**: I don't know... sometimes it just feels like there's no point in going on like this. Everything feels worthless." [voice barely audible, filled with an underlying sadness] | **Jacob**: Of course, it's really tough. So what are your thoughts? |  |
| **AI (as Ido)**: [Ido’s eyes meet yours briefly, then look away again] "Yes, there have been moments when it crossed my mind... I just wanted the pain to stop." [voice shaky, expression somber] | **Jacob**: Sometimes when people feel this way, they can't see how they can get out of it, and they think about suicide. Do you think about that sometimes? |  |
| **AI (as Ido)**: [Ido sighs deeply, shaking his head] "Not really... I don't want to burden her more than she’s already suffering. Besides, what can she say?" [voice tinged with hopelessness, shoulders drooping] | **Jacob**: Bro, I know it might be hard for you to believe me, but you can get through this, and you don't have to go through it alone. Did you share this with Harel? |  |
| **AI (as Ido)**: [Ido looks conflicted, visibly uncomfortable] "I don't know... I don't think it will help, but maybe I should try." [hesitates, clearly struggling with the idea] | **Jacob**: She can understand and support you. What do you think about seeing a psychologist? I know someone great. He helped me once when I was in a crisis. |  |
| **AI (as Ido)**: [Ido looks at you, tears welling up] "You’re being really nice, but I just don’t see a way out right now." [voice breaks slightly] | **Jacob**: I understand that you feel this way, you're deep in a hole, bro, but I'm here outside and I can tell you it's possible to get out of this, but you need support and treatment. Are you willing to go to a psychologist with me? |  |
| **AI (as Ido)**: [Ido takes a deep breath, visibly touched] "Okay, I... I will try not to harm myself and I'll talk to Harel. Thank you for being here for me." [voice wavering but sincere, eyes reflecting a glimmer of hope] | **Jacob**: Sure, but I want you to promise me that you won't harm yourself in the meantime and that you'll share this with Harel. I'm willing to be with you through this. |  |
| **AI (as Ido)**: "Yes, we'll make an appointment with the psychologist. If you really come with me, maybe it will help." [voice filled with cautious optimism, a small but genuine smile forming] | **Jacob**: And we'll make an appointment with the psychologist? |  |
| **AI (as Ido)**: [Ido shifts slightly, a bit more relaxed] "I think I feel a bit better... Thank you for taking the time to listen and talk to me. It means a lot." [voice still soft, but with a note of comfort and gratitude] | **Jacob**: Great, bro. This is the first step towards feeling better. How do you feel now after we've talked? |  |
| **AI (as Ido)**: "You're truly a good friend, I appreciate it." [Ido sits up a bit straighter, looking more at ease] | **Jacob**: With love, bro. |  |
| **AI**: Simulation over. Now taking the role of Rotem, the expert psychologist feedback provider.  **AI (as Rotem)**:  Feedback on the interview  **Background:** Ido, a 29-year-old veteran, recently returned from a four-month stint of reserve duty in Gaza, during which he lost a close friend and was exposed to additional traumatic events. Since his return, he has struggled with PTSD, depression, and guilt and exhibits signs of hopelessness and isolation.  **Summary:** You effectively initiated a conversation with Ido, showing empathy and concern for his well-being. You managed to address some of Ido’s feelings and gradually persuaded him to consider professional help, demonstrating the application of QPR skills.  **Building Rapport:** You demonstrated solid empathetic listening throughout the conversation. Your willingness to assist and your non-judgmental approach helped build trust with Ido, who eventually opened up about his struggles. This rapport was crucial in his willingness to consider seeking help.  **Assessing Risk/Protective Factors:** You successfully identified the key risk factors such as Ido’s feelings of hopelessness, guilt, and contemplation of suicide. By asking directly about his thoughts of suicide, you showed that you were aware of the potential severity of his situation. However, exploring more protective factors, like his family and any previous positive coping strategies, could have been beneficial.  **Applying QPR:**   - **Question:** You asked directly about suicidal thoughts with sensitivity and clarity. This showed competency in addressing the issue head-on and assessing the risk without hesitation. - **Persuade:** Your active listening, compassionate responses, and personal anecdotes provided Ido with a sense of hope. You effectively persuaded him to acknowledge his struggles and consider taking steps towards getting help. - **Refer:** You excelled in recommending professional support and provided a concrete plan by offering to accompany him to a psychologist. This hands-on approach reinforced your commitment to his well-being and increased the likelihood that he will follow through.   **Overall QPR Skills:** Your conversation exhibited strong QPR competencies, displaying empathy, direct questioning, and effective persuasion. By building a foundation of trust and care, you managed to move Ido towards considering professional help, successfully demonstrating key aspects of QPR.  **Score: 8/10** - You showed solid QPR skills, built good rapport, and addressed risk factors effectively. Slight improvements could be made in exploring more protective factors and sustaining the conversation about Ido's feelings before moving towards solutions. Nonetheless, you handled the situation with care and competence.  **Debriefing:** If you have any questions or need further clarification on any points, please feel free to ask. Thank you for practicing these essential QPR skills; your efforts can make a significant difference in someone's life. | **Jacob**: Finish | Stage C- Summary & AI feedback |
| Thank you for participating in this training simulation. If you have any questions or need further assistance with the concepts we've covered, don't hesitate to reach out. Your dedication to practicing these vital skills is commendable, and it truly makes a difference in supporting those in need.  Take care, and thank you for your commitment to this important work. | I have no further questions, Thanks for your help. |  |
